# The prebiotic effects of omega-3 fatty acid supplementation: A six-week randomised intervention trial

**DOI:** 10.1080/19490976.2020.1863133

**Published:** 2020-12-31

**Authors:** Amrita Vijay, Stuart Astbury, Caroline Le Roy, Tim D Spector, Ana M Valdes

**Affiliations:** a Department of Twin Research and Genetic Epidemiology, King’s College London, London, UK; b Division of Rheumatology, Orthopaedics and Dermatology, School of Medicine, University of Nottingham, Nottingham, UK; c Nottingham Digestive Diseases Centre, School of Medicine, University of Nottingham, Nottingham, UK; d NIHR Nottingham Biomedical Research Centre, Nottingham University Hospitals NHS Trust and the University of Nottingham, Nottingham, UK

**Keywords:** Dietary intervention, Omega 3, Fibre, Prebiotic, Gut microbiota

## Abstract

Prebiotics are compounds in food that benefit health *via* affecting the gut microbiome. Omega-3 fatty acids have been associated with differences in gut microbiome composition and are widely accepted to have health benefits, although recent large trials have been inconclusive. We carried out a 6-week dietary intervention comparing the effects of daily supplementation with 500 mg of omega-3 versus 20 g of a well-characterized prebiotic, inulin. Inulin supplementation resulted in large increases in *Bifidobacterium* and Lachnospiraceae. In contrast, omega-3 supplementation resulted in significant increases in *Coprococcus spp*. and *Bacteroides spp*, and significant decreases in the fatty-liver associated *Collinsella spp*. On the other hand, similar to the results with inulin supplementation which resulted in significant increases in butyrate, iso-valerate, and iso-butyrate (*p* < .004), omega-3 supplementation resulted in significant increases in iso-butyrate and isovalerate (*p* < .002) and nearly significant increases in butyrate (*p* < .053). *Coprococcus*, which was significantly increased post-supplementation with omega-3, was found to be positively associated with iso-butyric acid (Beta (SE) = 0.69 (0.02), *P* = 1.4 x 10^−3^) and negatively associated with triglyceride-rich lipoproteins such as VLDL (Beta (SE) = −0.381 (0.01), *P* = .001) and VLDL-TG (Beta (SE) = −0.372 (0.04), *P* = .001) after adjusting for confounders. Dietary omega-3 alters gut microbiome composition and some of its cardiovascular effects appear to be potentially mediated by its effect on gut microbial fermentation products indicating that it may be a prebiotic nutrient.

## Introduction

The human gut is home to trillions of bacteria forming a complex ecosystem that mediates host metabolic homeostasis.^1^Diet can shape the composition of the gut microbiota. It is therefore possible to also alter the metabolic signatures of the gut microbial populations^2–4^ by influencing the level and variety of substrates available for gut bacteria to metabolize.^5^ Although the majority of dietary components are directly absorbed in the upper digestive tract, some nutrients such as fiber remain undigested and are fermented in the large intestine and are commonly referred to as prebiotics. The breakdown of carbon sources, such a dietary fiber, by the gut microbiota leads to the production of short-chain fatty acids (SCFAs) and branched-chain fatty acids (BCFAs), ^6^ which in turn have been implicated in a variety of immunological, metabolic, and hormonal effects, such as promoting satiety, reducing inflammation, and improving glucose and lipid metabolism.^7,8^ Dietary fibers can modulate gut microbiota composition in through the enrichment of bacterial taxa that utilize the substrate and tolerate or benefit from the environmental changes caused by fiber fermentation.^9,10^ Therefore, the term ‘prebiotic’ has been recently revised to include ingredients that allow specific changes to not only the composition but also the activity of the gastrointestinal microflora that confers benefits upon host well-being and health.^11^

Many recent publications have documented the effects of prebiotic dietary fiber on health-related traits via their effects on the gut microbiome.^9,10,12^ On the other hand, the impact of dietary fats, such as omega-3 polyunsaturated fatty acids (PUFAs), on the gut microbiota is less well defined. Although studies have shown that the supplementation of omega-3 provides multiple health benefits against different chronic degenerative diseases, ^12–16^ recent large trials have been inconclusive for normal subjects.^17^ The role of the gut microbiome and individual variation in modulating these effects are yet to be explored. The influence of omega-3 on the composition of the gut microbiome has been previously explored via observational studies.^18^ Small randomized controlled trials that have found relatively small changes in the composition of the intestinal microbiome^19–21^ and the functional consequences, including the levels of SCFAs is a question that have been explored, but so far the latter has been done predominantly in animal models.^22–24^ Some recent human interventional studies have investigated the effects of high, pharmacological doses of EPA and DHA supplementation^21^ on gut microbiome composition the effect of omega-3 intake in doses compatible with dietary intake from food (as could correspond to eating oily fish two times per week) on both gut microbiome composition and SCFA/BCFA production are lacking. In this study, we have investigated the prebiotic potential of omega 3 compared to a well-characterized prebiotic, inulin fiber. The prebiotic potential of omega 3 was evaluated based on its effect on the gut microbiome composition and function (measured by associations of the composition of the gut microbiome with changes in short-chain fatty acids and lipid metabolites) at the end of a 6-week intervention.

## Methods

### Study population

Study subjects were enrolled from the TwinsUK registry, a national register of adult twins recruited as volunteers without selecting for any particular disease or trait traits. A total of 69 subjects were enrolled into the study and randomized into either the omega 3 or fiber arm.

### Study design and intervention

Participant eligibility included those aged >18 y who had a body mass index (BMI) between 20 and 39.9 kg/m2 and had a low habitual fiber consumption of less than 15 g/d. The following exclusion criteria were considered: ongoing or planned regular use of other omega-3 PUFA or cod liver oil supplements; seafood allergy; concomitant use of non-steroidal anti-inflammatory medications, including aspirin; current treatment for any chronic inflammatory condition or malignancy; previous colonic or small bowel resection; current smoker (minimum 6 months smoking cessation) and pregnancy. If an individual was eligible, he/she was consented and booked in for their baseline clinical visit at the clinical research facility at St Thomas’ Hospital, London, UK. Participants were randomized to take either 20 g of inulin fiber or 500 mg of omega-3 supplements daily (165 mg of EPA, 110 mg DHA, in gelatin capsules) for a period of 6 weeks. Neither participants nor researchers were blinded to the interventions and hence allocation order. The participants were booked in for a follow-up visit at the end of the 6-week intervention period. Randomization was performed using an online software (www.sealedenvelope.co.uk). All participants provided written informed consent. The trial was approved by the West Midlands Black Country Research Ethics Committee (18/WM/0066) and is registered under the clinicaltrials.gov database (NCT03442348).

### Sample collection

Blood, stool, and anthropometric measures (height, weight, blood pressure, body composition) were collected at both the baseline and follow-up visits. Blood samples were collected from participants between 8:30am and 10am during each visit. Participants were instructed to come in fasted state at least since 9 pm the night before (i.e. minimum fasting time was 11.5 hours). Blood samples were collected using Serum Separator Tubes (SST) and were processed within 2–3 hours of collection for separating serum and aliquoted for storage at −20 C until the end of the intervention period.

Diet and lifestyle patterns were measured at baseline, mid-intervention (i.e. 3 weeks into the intervention), and at follow-up using a set of validated questionnaires including the EPIC-Norfolk: Food Frequency Questionnaire;^25^ the Bristol stool form scale^26^, and the SF-12 quality of life questionnaire.^27^ Fecal samples were provided at study visits and immediately frozen at −80°C until DNA extraction, which occurred as soon as the study was completed.

### Microbiota analysis

The stool DNA extraction is detailed in Goodrich et al.^28^ of 100 mg were taken from the sample and used for extraction. There was no homogenization prior to this step. Fecal samples were collected and the composition of the gut microbiome was determined by 16 S rRNA gene sequencing carried out as previously described.^29,30^

Briefly, the V4 region of the *16S rRNA* gene was amplified using universal primers 355 F (CCAGACTCCTACGGGAGGCAGC) and 806 R (GGACTACHVGGGTWTCTAAT). Amplified DNA was sequenced on the MiSeq platform (Illumina). Read filtering and clustering were carried out using the MYcrobiota pipeline.^31^ Chimeric sequences were filtered using the VSEARCH algorithm within Mothur, and reads were clustered into OTUs using closed-reference clustering against the SILVA database v132 based on a 97% similarity. Diversity metrics (Shannon index, observed OTUs, and Unweighted UniFrac) were calculated by rarefying the OTU table down to 7000 sequences per sample 50 times and taking the average. These analyses were carried out in QIIME 2 (v2018.11).

### Metabolite analysis

Serum short and branched chain fatty acids:

The method employed for the serum SCFA and BCFA was based on the in-situ pentafluorbenzylation of the free acid species, followed by GC-NCI-MS determination of the resulting derivatives. Measures were only obtained from serum and not feces given recent reports indicating that circulating levels but not fecal levels, in much larger sample sizes, correlate with clinical traits.^32^ All reagents and primary standards for acetic, propionic, iso-butyric, butyric, isovaleric, valeric acids including the internal standard (d4 acetic acid) were purchased from Sigma Aldrich.100 µl of serum were dispensed into an Eppendorf tube, followed by the addition of 100 µl of acetonitrile containing internal standard (d4 acetic acid) at 6 µmol/l. After vortexing for 30s, the mixture was treated with 5 µl of neat pentafluorobenzyl bromide, followed by 3 µl of neat diisopropylethylamine and then incubated for 30 min at 60°C to effect derivatization. On cooling, 100 µl of heptane were added and the mixture vortexed briefly to facilitate analyte transfer. Upon a brief centrifugation, the supernatant (containing the pentafluorbezyl derivatives) was transferred to a glass insert vial for GC-MS analysis. The analysis was performed on an Agilent Technologies 6890 gas chromatograph interfaced with a 5973 mass spectral detector operated in negative chemical ionization mode. The system inlet was operated in splitless mode with the injection point temperature set at 220°C. The short-chain fatty acid derivatives were separated on a DB-5 MS capillary column of dimensions 30 m × 250 µm ×0.25 µm using temperature programming and a constant carrier flow rate. Mass spectral data were acquired in selected ion monitoring mode with masses corresponding to the carboxylate anion chosen for both qualification and quantitation. Five level calibration curves were generated by preparing standards spanning the concentration range of interest and running under identical conditions. Briefly, a binary stock solution containing acetic acid and propionic was prepared at concentrations of 1.5 mg/ml and 0.15 mg/ml, respectively. Similarly, a stock solution containing iso-Butyric, Butyric, iso-Valeric, and Valeric was prepared with concentrations of 0.15 mg/ml, 0.27 mg/ml, 0.04 mg.ml and 0.15 mg/ml, respectively. It was necessary to have acetic/propionic calibrators independent of the other species to prevent traces of the other species present contributing to the lower levels of butyric onwards. These two independent stock mixtures were then diluted to form working calibrators which in turn were serially diluted to form the basis of the calibration. Hundred-microliter aliquots of the resulting calibrants were prepared as per serum.

Lipids and gut-derived metabolites:

Circulating levels of cholesterol and triglyceride fractions from fasting serum samples were measured using the high-throughput 1 H-NMR metabolomics platform (Nightingale Health Ltd., Helsinki, Finland; nightingalehealth.com/).^33^ In addition, circulating levels of Docosahexaenoic acid (DHA) and Total omega 3 were also measured using the same platform.

Certain gut-derived metabolites such as TMAO and IPA were measured using tandem mass spectrometry with the Biocrates MxP Qaunt 500 kit (Biocrates Life Science AG, Innsbruck, Austria).^34^

### Statistical analysis

OTUs with a relative abundance of <0.1% in every sample were removed, and zero inflated relative OTU abundances were inverse normal transformed before further analysis. We investigated the different effects of fiber and omega-3 interventions on changes in OTU abundances (genus level) by running general linear models, with change in OTU abundance as the outcome and fiber/omega-3 intervention as the exposure. We adjusted for age, gender, and BMI and multiple testing using false discovery rate (FDR<0.05). Linear regressions were employed to investigate the association between OTUs and serum metabolites adjusting for covariates such as age, sex, BMI, and multiple testing (FDR < 0.05). All statistical analyses were carried out in R v3.5.2.

## Results

Sixty-nine participants were randomized into either the omega-3 or inulin fiber intervention arms. The descriptive characteristics of study participants are shown in Table 1.

**Table 1.** Descriptive characteristics of participants in the fiber and omega 3 intervention arms at baseline and follow-up, respectively, both interventions were well tolerated with no major adverse events (AEs) reported. No differences in measures of habitual diet as assessed by FFQ were observed in either arm between baseline and follow-up (not shown)

	Fiber intervention		Omega 3 intervention	
Variable	Baseline (± SD)	Follow-up (± SD)	Baseline (± SD)	Follow-up (± SD)
Men/women (%)	5/30 (14.3%/85.7%)	-	3/34 (8.8%/91.1%)	-
Age (y)	66.83 ± 9.3	-	63.67 ± 10.76	-
BMI (kg/m2)	26.68 ± 4.4	26.64 ± 4.31	27 ± 3.740	26.40 ± 4.945
Acetic acid (µmol/l)	43.43 ± 68.69	49.10 ± 104.00	174.2 ± 127.8	180.3 ± 171.4
Propionic acid (µmol/l)	10.13 ± 0.79	11.97 ± 5.62	9.71 ± 5.96	10.48 ± 7.52
Butyric acid (µmol/l)	8.39 ± 0.73	11.61 ± 4.85	7.06 ± 4.544	7.78 ± 3.52
Valeric acid (µmol/l)	7.21 ± 0.51	10.18 ± 0.42	0.99 ± 0.606	1.183 ± 0.586
Iso-butyric acid (µmol/l)	10.69 ± 5.70	14.3 ± 6.82	8.47 ± 5.75	10.89 ± 5.42
Iso-valeric acid (µmol/l)	7.13 ± 2.70	8.12 ± 5.11	6.94 ± 3.80	9.83 ± 3.20
Serum cholesterol (mmol/l)	5.136 ± 1.030	4.347 ± 1.066	5.207 ± 0.883	4.717 ± 0.558
XL-VLDL-C (mmol/l)	0.004 ± 0.006	0.004 ± 0.006	0.006 ± 0.007	0.003 ± 0.005
VLDL-TG (mmol/l)	0.558 ± 0.293	0.541 ± 0.333	0.617 ± 0.309	0.488 ± 0.316

### Diversity indices

**Figure 1.**
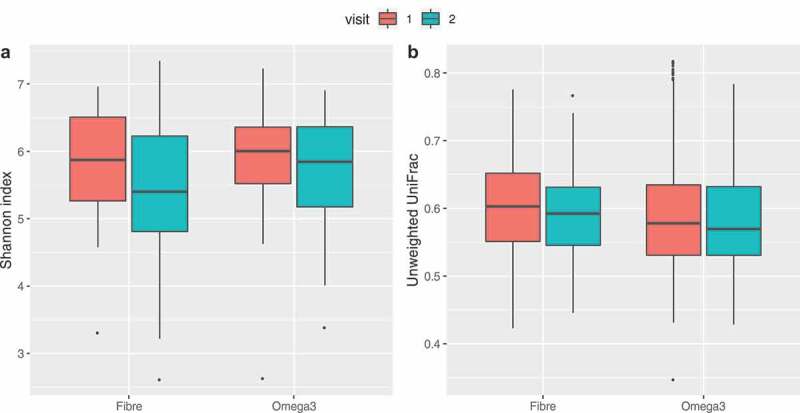
(a) Difference in Shannon α diversity index between inulin fiber and omega-3 at baseline and follow up (*p* = .62). (b) Differences in beta diversity index between inulin fiber and omega-3 at visit 1 (baseline) and visit 2 (follow up). *P*-values assessed by paired t-test

No significant change between baseline and follow-up was observed in average alpha (both Shannon and observed OTUs) and beta diversities for omega-3 and inulin intervention arms as shown in Figure 1.

### OTUs and serum metabolites associated with both treatment arms

**Figure 2.**
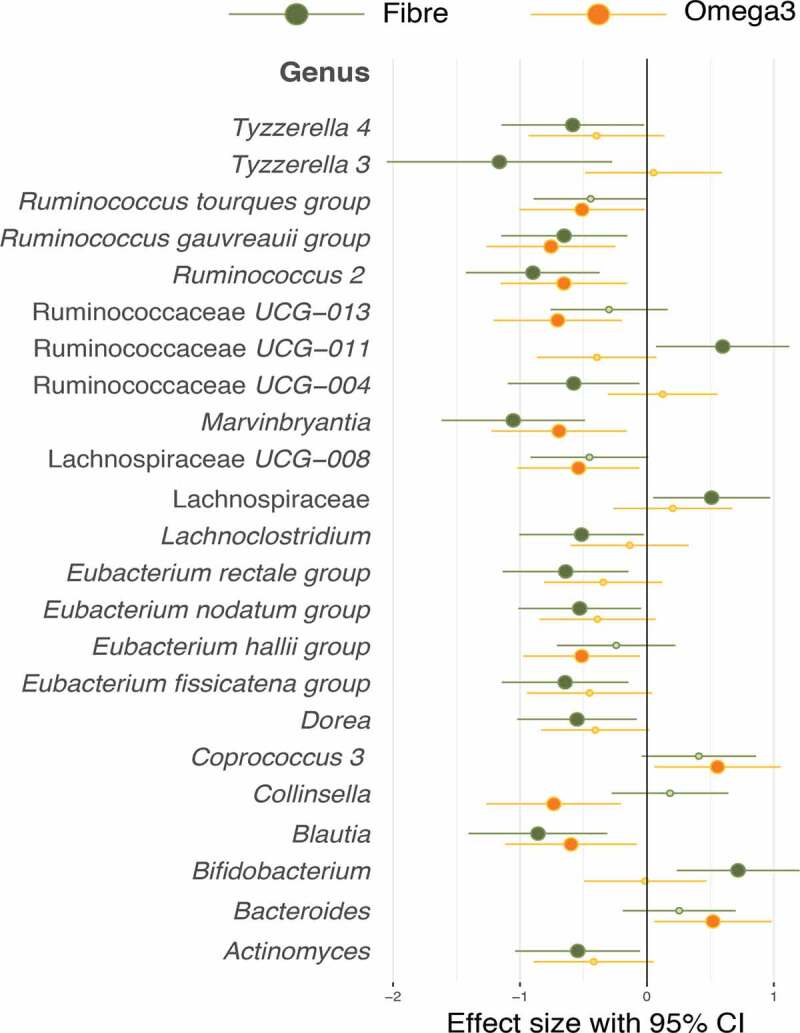
Forest plot of effect sizes with 95% confidence intervals showing association of all significant (FDR adjusted *p* value <.05) OTUs in the Fiber and omega-3 intervention arms. Smaller dots on the fiber and omega-3 arm indicate a loss of statistical significance. Association was tested by paired t-tests between baseline and follow-up

Most significantly associated with omega-3 supplementation were *Coprococcus* and *Bacteroides*, whereas *Bifidobacterium, Ruminococcaceae* UCG-011 and an unidentified taxon belonging to a genus from the *Lachnospiraceae* family was found to be significantly increased in the fiber group. There were certain species of the Lachnospiraceae related genus which were also increased in the omega-3 group, however this did not reach statistical significance as shown in Figure 2.

In order to assess if the relatively small changes in the intestinal microbiome that we observed at the end of the 6-week intervention period had functional consequences, we measured gut microbiome-derived metabolites. There were significant increases in the levels of certain SCFAs and BCFAs in both the fiber and omega-3 arms with fiber eliciting a greater effect on SCFAs and BCFAs increase (Table 2). However, no significant differences were seen in the levels of TMAO and IPA in either of the arms. The levels of circulating docosahexaenoic acid (DHA) and FAW3 total omega-3 as a proportion of total fatty acids (FA) shows a significant increase in the omega-3 arm.

**Table 2.** Changes in the levels of serum metabolites in both arms (*p*-values from paired t-tests)

	Fiber		Omega-3	
Metabolite	Follow up-Baseline	p value	Follow up-Baseline	p value
Acetic acid (μmol/l)	5.67	0.5213	11.42	0.77
Propionic acid (μmol/l)	1.85	0.1418	1.12	0.081
Butyric acid (μmol/l)	3.23	0.0004***	1.88	0.053
Valeric acid (μmol/l)	2.97	0.3028	0.2	0.21
Iso-butyric acid (μmol/l)	3.61	0.0038**	2.69	0.002**
Iso-valeric acid (μmol/l)	0.99	0.001*	1.08	0.001*
TMAO (μmol)	1.32	0.0832	1.12	0.122
IPA (μmol)	0.89	0.112	0.42	0.142
DHA/total fatty acids (μmol)	0.257	0.0932	1.230	4.06E-05***
Total omega-3/total fatty acids	−0.2005	0.1387	1.174	4.06E-05***

### Association of the gut microbiome composition with short-chain fatty acids and cardiovascular markers

**Figure 3.**
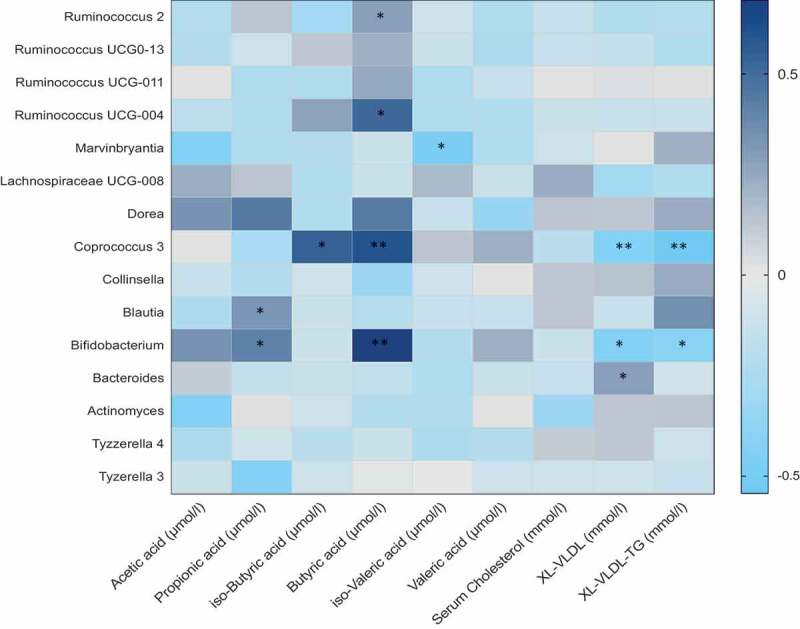
Heat map showing OTUs clustered at genus level and their association with serum metabolites. Values are beta coefficients from linear models adjusted for BMI, age and gender. The heat map is color coded by correlation according to the table legend (dark blue for positive and light blue for negative correlations). *p* values are adjusted for FDR and are indicated as FDR *p* < .05 (*) *p* < .01 (**)

We then looked at the association between all genera that were significantly altered in one of the two interventions and SCFA, BCFA, and serum metabolites. We observed that in most cases, genera positively associated with SCFAs or BCFAs were also negatively associated with serum lipids as shown in Figure 3. For instance, the relative abundance of *Coprococcus*, that was significantly increased in the omega-3 arm at follow-up compared to baseline, was found to be positively associated with iso-butyric acid (Beta (SE) = 0.69 (0.02), *P* = 1.4 x 10^−3^). In addition to these, the other genera that were increased in the omega-3 arm such as *Ruminococcaceae UCG-004*, showed positive significant associations with SCFAs such as butyrate (Beta (SE) = 0.67 (0.04), *P* = 1.1 x 10^−3^) and valerate (Beta (SE) = 0.62 (0.02), *P* = 1.3x 10^−3^) respectively. The genera *Bifidobacterium*, one of the genera which was significantly increased in the inulin fiber arm only was positively associated with butyrate (Beta (SE) = 0.780 (0.02), *P* = 1.5 x 10^−5^). In addition to the associations with short-chain fatty acids, we associated the different genera with markers of cardiovascular disease. We found that *Coprococcus* was negatively associated with VLDL (Beta (SE) = −0.381 (0.01), *P* = .001), VLDL-TG (Beta (SE) = −0.372 (0.04), *P* = .001). The genera *Bifidobacterium* was also negatively associated with XL-VLDL and VLDL-TG (Beta (SE) = −0.472 (0.05), *P* = .002; Beta (SE) = −0.463 (0.03), *P* = .001) respectively.

### Effect of BMI on gut microbiome composition and serum metabolite levels

Since the study included obese participants, we carried out sub-analyses stratifying obese (BMI>30) and non-obese subjects (BMI<30) (Supplementary Table 1). We found no significant differences in the composition of the gut microbiome between the two groups except for specific bacterial species such as *Coprococcus 3* which was marginally significant in the high BMI/obese group (>30) in the omega 3 arm (*p* = .04). Similarly, we saw a significant association of *Bifidobacterium* in low BMI/lean group in the fiber arm only (*p* = .02). However, we found no significant associations of BMI with SCFAs, BCFAs, and cardiovascular markers (Supplementary Table 1)

## Discussion

In this study, we report small, consistent changes in the human intestinal microbiome associated with 6-weeks of supplementation with 500 mg of omega-3 FA and we compare them to changes seen with inulin fiber supplementation for the same length of time. There were significant changes in the levels of bacterial fermentation products following a 6-week intervention with omega-3 supplementation and the overall effects were comparable to inulin fiber supplementation supporting the role of omega-3 as a potential prebiotic.

As expected for inulin supplementation, we observed large and significant increases in *Bifidobacterium* and *Lachnospiraceae* and in butyrate production. In the case of Omega-3, the largest increases were in *Coprococcus* and *Bacteroides*. An increase in *Coprococcus* abundance due to omega-3 supplementation was also reported by Waston and coworkers^21^ using a much larger dose of omega-3 (4 g). In that study, significant increases in the abundance of *Bifidobacterium* were found, which we failed to observe (see Figure 1) suggesting that this effect may be dose dependent. However, in both cases, we see significant drops in the relative abundance of some SCFA producing bacteria, such as *Eubacterium* and some types of *Ruminococaccae*. This is consistent with what is known about how prebiotics affect bacterial communities.^35^ Different substrates increase the relative abundance of different species and this in turn, results in decreases of other species, some of which may also be SCFA producers or be involved in some of the health benefits linked to the gut microbiome. This suggests that optimal prebiotic supplementation strategies should focus on feeding bacterial communities and requires an understanding of the interdependencies between bacterial strains, rather than the increase of a single carbon source.

The lack of significant change in microbial diversity associated with omega-3 PUFA intervention is consistent with previous studies, in which there was either no change, or a small change in α diversity.^21^ However, the current study highlights significant shifts in the composition of the gut microbiome with specific short-chain fatty acid-producing bacteria that increased in not only the fiber group but also in the omega-3 group.

Emerging data have demonstrated that an aberrant gut microbiota composition is associated with several diseases, including metabolic disorders. One of the mechanisms by which the microbiota affects human health and disease is its capacity to produce metabolites which are either associated with the development of disease or those that protect against disease. One such versatile class of microbial metabolites are short and branched chain fatty acids that are commonly produced from the microbial fermentation of dietary fibers and are likely to have broad impacts on various aspects of host physiology.^8^ In this study, we observed significant differences in the levels of short and branched chain fatty acids which were found to be positively associated with specific SCFA-producing bacteria. *Coprococcus* was significantly increased post-supplementation with Omega-3. The increase in *Coprococcus* was positively associated with iso-butyric and butyric acid levels which are fatty acids that are produced by the breakdown of amino acids rather than the breakdown of carbohydrates and are also referred to as branched-chain fatty acids.^36^ The association of *Coprococcus* with butyrate has been well established in previous studies, ^37,38^ however, its association with iso-butyrate has not been previously reported. The results of the correlation analysis suggested significant positive associations of the genus *Coprococcus* with iso-butyric and butyric acid production. We note however, that although the increase of *Coprococcus* in the omega-3 arm was more evident among obese individuals than among non-obese individuals, we found no corresponding difference in levels of SCFAs or other markers. Several studies support the role of both the microbiota and n-3 PUFAs in regulating inflammatory, cardiovascular, and immune markers.^8,10,12,39,40^ In the current study, we report negative associations of *Coprococcus* with triglyceride-rich lipoproteins such as VLDL and VLDL-TG. The current findings suggest that the cardiovascular benefits of omega 3 supplementation may be mediated by the gut microbiome, however this requires further investigations along with the potential differences of *Coprococcus* effects on health among obese and non-obese individuals.

Previous larger scale trials such as ASCEND and REDUCE-IT; which tested the role of omega-3 on reducing cardiovascular events have generated conflicting results.^41,42^ Although the results from these studies highlighted that the cardio-metabolic effects could be dependent on dosage of omega-3 supplementation and cardiovascular risk score of the subjects, ^43^ the role of the gut microbiome interacting with omega-3 could also be a crucial factor that could result in the variability observed. Interestingly, we also find that omega-3 supplementation results in a strong significant decrease in the relative abundance of the genus *Collinsella* which we have recently reported to be increased by 3-fold in individuals with non-alcohol fatty liver disease.^44^ Given that NAFLD is known to be a risk factor for both insulin resistance and cardiometabolic disease this suggests an important potential microbiome pathway by which omega-3 has a positive effect on health.

We acknowledge several limitations in our study. Firstly, the trial lacked direct comparisons to a placebo arm; however, the prebiotic effect of omega-3 was compared to inulin fiber, a well-characterized prebiotic. Secondly, the participants were predominantly female and therefore our results may not generalize to diverse populations. This may have also had some effects on our results in relation to the gut microbiome; however, the effect of gender has been adjusted for in all statistical analyses. Thirdly, although it has been shown that over 90% of microbes are associated with a vast proportion of the measured gut metabolites (>80%), the effects of these associations on host health are mainly derived from microbial metabolic pathways that are shared amongst microbial communities interacting with their surrounding environment.^45^ Therefore a combined approach of metagenomics and metabolomics may provide a better understanding of the functional role of microbial species and communities in mediating immune and cardiovascular benefits. Lastly, although circulating plasma SCFA levels are more directly linked to metabolic health, they may not truly reflect the levels of SCFAs produced and absorbed by the gut.^46^ In addition, the complexity and challenges faced by the handling of these volatile molecules adds the uncertainty of replicating levels from both sources.^47^ Therefore, our results apply solely to the effect of gut microbes on circulating levels of SCFAs and BCFAs and we cannot extrapolate our conclusions to fecal levels of these compounds. Based on the current findings, we suggest that we can consider omega-3 fatty acids to possess the functional properties of a prebiotic due to the fact that they not only have the potential to induce small changes in the composition of the gut microbiome but also increase the levels of certain gut-derived metabolites such as BCFAs and SCFAs that have shown to positively impact metabolic health.^48^ Although previous studies have shown increased butyrogenic capacity and therapeutic effects on trialing relatively large doses of omega 3 (greater than 4 g/d), ^21,49,50^ these doses could be challenging to achieve through a normal diet.^51^ However, the current study has highlighted that prebiotic effects of omega 3 can be achieved by taking as little as 500 mg of EPA + DHA daily for 6 weeks. Furthermore, observational and clinical trials have widely demonstrated the potential benefits of prebiotics on human health^52^ and therefore the next steps to improve public health in the context of non-communicable diseases would be to test combinations of prebiotics to target specific diseases.

## Supplementary Material

Supplemental Material
