# Supplementary material for: The prebiotic effects of omega-3 fatty acid supplementation: A six-week randomised intervention trial
Source: Gut Microbes. 2020 Dec 31;13(1):1863133. doi: 10.1080/19490976.2020.1863133 (PMC7781624; doi:10.1080/19490976.2020.1863133)
Supplement: Supplemental Material [file KGMI_A_1863133_SM1776.zip › supplement/Supplementary tables_Omega 3.docx]

Supplementary tables:

**Table 1**: Analysis of significant OTUs and serum metabolites based on BMI <30 and >30 in both fibre and omega 3 intervention groups

|  | **Fibre intervention** | | | | **Omega 3 intervention** | | | |
| --- | --- | --- | --- | --- | --- | --- | --- | --- |
|  | **BMI<30 (N=28)**  **OR (lower-upper 95% CI)** | **P value** | **BMI>30 (N=7)**  **OR (lower-upper 95% CI)** | **P value** | **BMI<30 (N=26)**  **OR (lower-upper 95% CI)** | **P value** | **BMI>30 (N=6)**  **OR (lower-upper 95% CI)** | **P value** |
| Ruminococcaceae UCG-011 | 0.58 (0.33-1.01) | 0.05 | 0.37 (0.07- 1.96) | 0.24 | 0.80 (0.48- 1.29) | 0.35 | 0.33 (0.06-1.60) | 0.17 |
| Lachnospiraceae | 1.54 (0.91-2.60) | 0.105 | 0.76 (0.12-4.59) | 0.76 | 1.06 (0.68- 1.66) | 0.78 | 2.40 (0.60- 9.67) | 0.21 |
| Coprococcus 3 | 0.69 (0.85- 2.12) | 0.12 | 0.32 (0.04-2.20) | 0.25 | 0.68 (0.41- 1.13) | 0.14 | 0.02 (0.003 -0.95) | **0.04** |
| Bifidobacterium | 1.80 (1.09- 2.30) | **0.02** | 5.20 (0.99- 27.28) | 0.05 | 1.02 (0.60-1.72) | 0.93 | 0.75 (0.18- 3.10) | 0.70 |
| Bacteroides | 1.34 (0.85- 2.12) | 0.20 | 0.76 (0.11-5.12) | 0.78 | 1.64 (1.0-2.69) | **0.04** | 2.56 (0.60-11) | 0.20 |
| Acetic acid (μmol/l) | 0.24  (0.11- 1.91) | 0.70 | 0.12  (0.08- 3.18) | 0.33 | 0.75  (0.13-2.40) | 0.90 | 0.57 (0.22- 2.18) | 0.72 |
| Propionic acid (μmol/l) | 1.44  (1.10-3.62) | 0.87 | 1.29  (0.11- 3.04) | 0.31 | 0.58  (0.07-1.56) | 0.32 | 0.28 (0.09- 3.18 | 0.43 |
| Iso-butyric acid (μmol/l) | 0.85  (0.76-2.43) | 0.57 | 0.75  (0.21- 1.80) | 0.60 | 1.22  (1.14-2.57) | 0.92 | 0.87  (0.32-1.08) | 0.91 |
| Butyric acid (μmol/l) | 1.91  (0.67-3.31) | 0.32 | 1.13  (0.33- 2.66) | 0.50 | 0.73  (0.43-0.91) | 0.67 | 0.44  (0.25-1.09) | 0.51 |
| Iso-valeric acid (μmol/l) | 0.30  (0.10-0.51) | 0.30 | 0.21  (0.08- 0.56) | 0.34 | 0.75  (0.10-0.96) | 0.66 | 0.87  (0.11-1.01) | 0.40 |
| Valeric acid (μmol/l) | 0.37 (0.18-2.06) | 0.14 | 0.51 (0.13-2.08) | 0.31 | 0.51 (0.31-1.28) | 0.40 | 0.47 (0.14-1.19) | 0.93 |
| Serum cholesterol (mmol/l) | 1.26  (0.07-2.11) | 0.74 | 1.06  (0.02-1.41) | 0.31 | 0.719  (0.13-1.98) | 0.14 | 0.64  (0.33-1.23) | 0.52 |
| Total cholesterol (mmol/l) | 0.71  (0.03-1.04) | 0.76 | 0.13  (0.02-1.02) | 0.98 | 0.11  (0.08-0.15) | 0.25 | 0.01  (0.005-0.07) | 0.66 |
| XL-VLDL (mmol/l) | 0.21  (0.03-1.02) | 0.70 | 0.18  (0.04-0.87) | 0.86 | 0.83  (0.26-1.85) | 0.80 | 0.52  (0.27-1.04) | 0.62 |
