# Supplementary figures and images for: The prebiotic effects of omega-3 fatty acid supplementation: A six-week randomised intervention trial
Source: Gut Microbes. 2020 Dec 31;13(1):1863133. doi: 10.1080/19490976.2020.1863133 (PMC7781624; doi:10.1080/19490976.2020.1863133)

## Slide 1
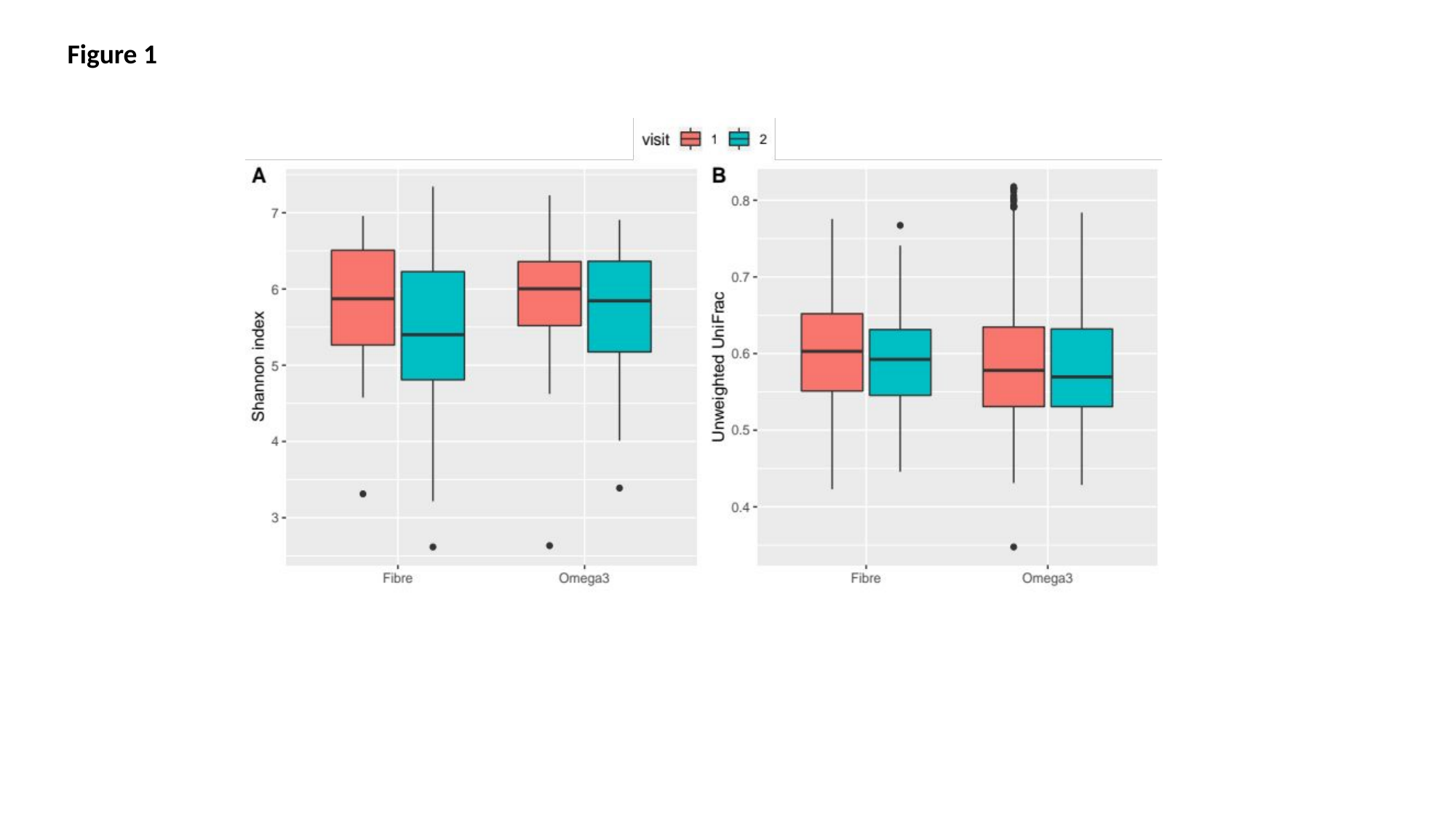

Figure 1

## Slide 2
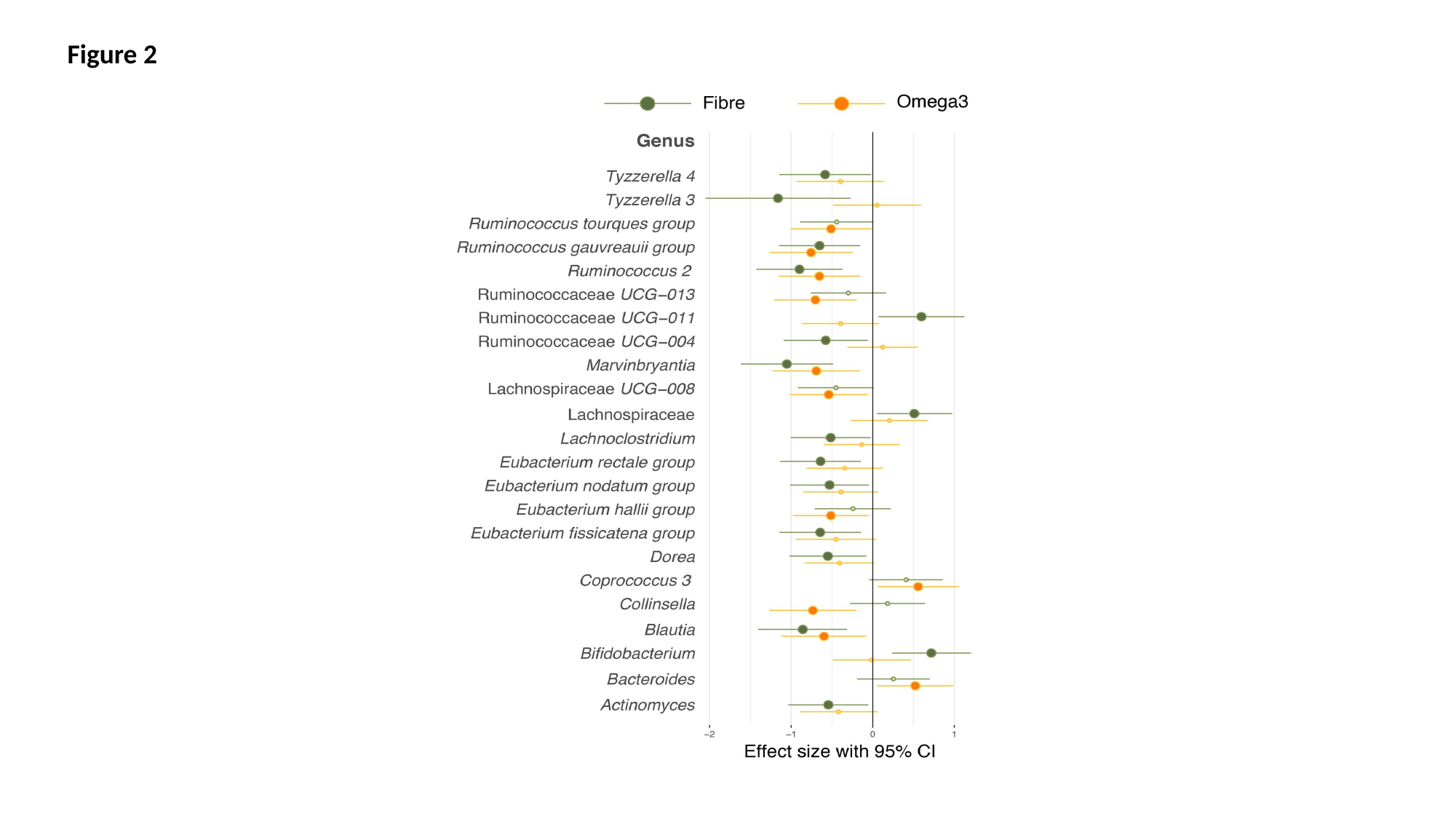

Figure 2

## Slide 3
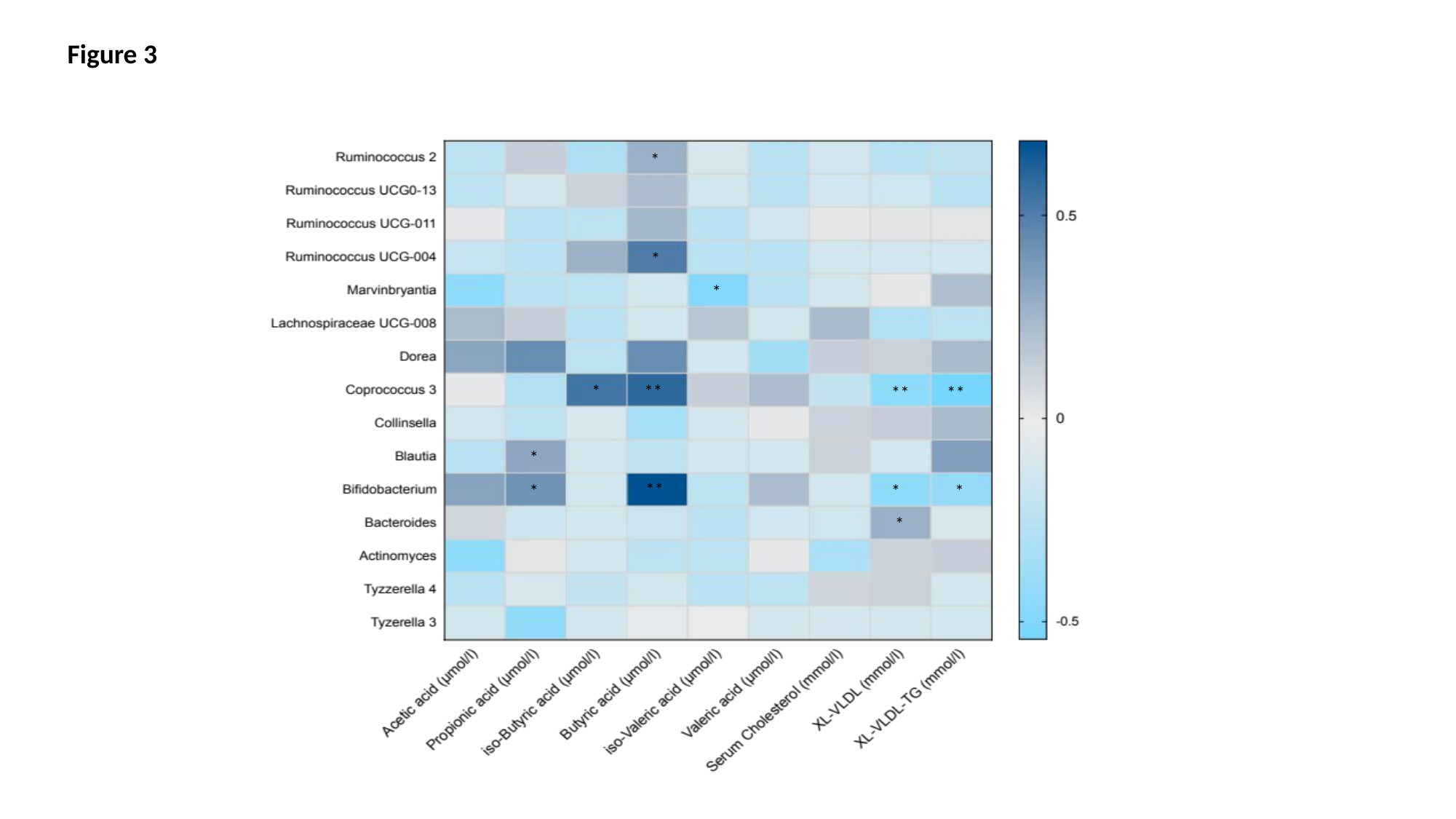

Figure 3
*
*
*
*
**
**
**
*
**
*
*
*
*

Supplement: Supplemental Material [file KGMI_A_1863133_SM1776.zip › supplement/Prebiotic effects of omega 3_all figures_111020.pptx]
